# Stochastic Analysis of Predator–Prey Models under Combined Gaussian and Poisson White Noise via Stochastic Averaging Method

**DOI:** 10.3390/e23091208

**Published:** 2021-09-13

**Authors:** Wantao Jia, Yong Xu, Dongxi Li, Rongchun Hu

**Affiliations:** 1 School of Mathematics and Statistics, Northwestern Polytechnical University, Xi’an 710072, China; 2 College of Big Data Science, Taiyuan University of Technology, Taiyuan 030024, China; dxli0426@126.com; 3 MIIT Key Laboratory of Dynamics and Control of Complex Systems, Department of Engineering Mechanics, Northwestern Polytechnical University, Xi’an 710129, China; rongchun_hu@nwpu.edu.cn

**Keywords:** statistical responses, predator saturation, predator competition, stochastic averaging method, stationary PDF, combined Gaussian and Poisson white noise

## Abstract

In the present paper, the statistical responses of two-special prey–predator type ecosystem models excited by combined Gaussian and Poisson white noise are investigated by generalizing the stochastic averaging method. First, we unify the deterministic models for the two cases where preys are abundant and the predator population is large, respectively. Then, under some natural assumptions of small perturbations and system parameters, the stochastic models are introduced. The stochastic averaging method is generalized to compute the statistical responses described by stationary probability density functions (PDFs) and moments for population densities in the ecosystems using a perturbation technique. Based on these statistical responses, the effects of ecosystem parameters and the noise parameters on the stationary PDFs and moments are discussed. Additionally, we also calculate the Gaussian approximate solution to illustrate the effectiveness of the perturbation results. The results show that the larger the mean arrival rate, the smaller the difference between the perturbation solution and Gaussian approximation solution. In addition, direct Monte Carlo simulation is performed to validate the above results.

## 1. Introduction

The Lotka–Volterra (LV) model [1] is a classic model for describing the interaction between the prey and predator in ecosystems with predator–prey relations [2,3]. Since the introduction of this model, several improved LV models have been investigated with consideration of different factors. Among those studies, the ones considering predator saturation and predator competition terms, respectively, have been introduced to characterize two special cases of the evolution of the ecosystems. The model with the predator saturation term describes a case where the preys are abundant, while the one with the predator competition term considers another case in which the predator population is very large compared with the prey population. The dynamics of these two models have been discussed in [1,4]. 

Moreover, the evolution of real-world biological systems usually suffers from unavoidable random perturbations in the natural environment [5,6,7,8,9]. Many investigations on the dynamics of predator–prey models with predator saturation and predator competition terms excited by stochastic perturbations have been reported [8,9,10,11,12,13,14]. In these studies, the stochastic perturbation is introduced as continuous stochastic excitations to disturb the birth rate of the prey and the death rate of the predator.

Nevertheless, in the environmental fluctuations, there are some drastic changes, including floods, earthquakes, tornados, or forest fires, which indicates that the evolutions of the ecosystem can be affected by unavoidable, sparse, discrete random jumps. Consequently, it is not appropriate to characterize the environmental effects by using only continuous random excitations. The combinations of continuous random processes and random jumps are viewed as more appropriate model for this case [15,16,17,18]. The ecosystem models excited by stochastic excitations with random jumps have been attracting increasing interests, and many reliable results have been obtained. In these studies, the Lévy process or jump-diffusion processes are commonly used in mathematical models to describe environmental perturbations with random jumps [19,20,21,22]. Most of them focus on the mathematical properties of the ecosystems, including the uniqueness, stability of the solutions, etc. [23,24,25,26,27]. However, for the prediction of the PDFs of the species populations, only the ecosystems enforced by stochastic processes with jumps have been investigated [28,29]. Therefore, more work needs to be done on deriving the stochastic responses of the ecosystems under stochastic excitations with random jumps.

The stochastic averaging method is an effective tool for investigating nonlinear systems excited by stochastic forcings. It was first applied to nonlinear systems under Gaussian white noise excitation [30,31,32], and then generalized to nonlinear systems with other types of stochastic excitations [33,34]. It has been successfully employed to study the stationary PDFs [6,7,8,9,11,12,28,35] and the optimal control [36] of species populations in ecosystems with small self-competition and small stochastic excitation. However, the solution of the ecosystem dynamics under continuous and random jump excitation by the stochastic average method has not yet been reported.

Inspired by this, here, we study predator–prey models with predator saturation and predator competition terms excited by combined Gaussian and Poisson noises. The governing equation for the solution of the stochastic model is approximately derived by applying the stochastic averaging method. Then, the statistical responses including the approximate PDFs and moments of the population densities are derived by solving the governing equation with a perturbation technique. Further, the effects of the system parameters as well as the stochastic excitations on the stationary responses are presented. The rest of the paper is organized as follows. Section 2 introduces two ecosystem models excited by combined Gaussian white noise and Poisson white noise and the averaged generalized Fokker–Planck–Kolmogorov (GFPK) equation. In Section 3, the effects of the parameters on the stochastic dynamics of the ecosystem are discussed. Finally, the conclusions are summarized in Section 4.

## 2. The Models

### 2.1. The Deterministic Models

In the present paper, we start from two deterministic prey–predator type ecosystems, one with an abundant prey population and the other with a large predator population, which can be viewed as generalizations of the LV model [1]. Although the two models are given based on different assumptions, fortunately we can define some new parameters to obtain a more general model that contains the two models as its special cases [8,37].

**Case 1**: prey population is abundant

Consider the case where the prey is abundant. The model is given as

(1) $$\begin{array}{l} {\dot{x}}_{1}=ax_{1}-sx_{1}^{2}-\frac{bx_{1}x_{2}}{1+Ax_{1}},\\ {\dot{x}}_{2}=-cx_{2}+\frac{fx_{1}x_{2}}{1+Ax_{1}}. \end{array}$$

where $x_{1}$ and $x_{2}$ are the prey and predator population densities, respectively; a and c represent the birth rate of the prey and the death rate of the predators. $-sx_{1}^{2}$ reveals the self-competition between the preys. The terms $\frac{bx_{1}x_{2}}{1+Ax_{1}}$ and $\frac{fx_{1}x_{2}}{1+Ax_{1}}$ in Equation (1) describe the interaction between the species [37]. These two interaction terms are proportional only to the predator population $x_{2}$, which implies that the consumption of the prey depends on the predator population and not on the prey population.

Model (1) can be reformulated in the following form

(2) $$\begin{array}{l} {\dot{x}}_{1}=x_{1}\left[\bar{a}-\bar{b}x_{2}-\frac{s}{\bar{f}}\left(-c+\bar{f}x_{1}\right)+g_{1}\left(x_{1},x_{2}\right)\right],\\ {\dot{x}}_{2}=x_{2}\left[-c+\bar{f}x_{1}+g_{2}\left(x_{1},x_{2}\right)\right]. \end{array}$$

with

(3) $$g_{1}\left(x_{1},x_{2}\right)=\frac{\bar{b}}{\bar{f}}Ax_{2}\frac{\bar{f}x_{1}-c}{1+Ax_{1}},g_{2}\left(x_{1},x_{2}\right)=-Ax_{1}\frac{\bar{f}x_{1}-c}{1+Ax_{1}}$$

by introducing

(4) $$\bar{f}=f-cA,\;\bar{a}=a-\frac{sc}{\bar{f}}=a-\frac{sc}{f-cA},\;\bar{b}=\frac{\bar{f}b}{\bar{f}+cA}=\frac{b}{f}\left(f-cA\right).$$

At this time, Model (2) has two equilibrium points, one unstable point $\left(0,0\right)$ and one asymptotically stable point $\left(\bar{c}/\bar{f},\bar{a}/\bar{b}\right)$.

**Case 2**: the predator population is large

When the population predator is large relative to the prey population, the prey–predator system can be modeled as

(5) $$\begin{array}{l} {\dot{x}}_{1}=ax_{1}-sx_{1}^{2}-\frac{bx_{1}x_{2}}{1+Bx_{2}},\\ {\dot{x}}_{2}=-cx_{2}+\frac{fx_{1}x_{2}}{1+Bx_{2}}. \end{array}$$

In this case, the prey consumption depends only on the prey supplement. Compared with model (1) in **Case 1**, Model (5) has different interaction terms $\frac{bx_{1}x_{2}}{1+Bx_{2}}$, $\frac{fx_{1}x_{2}}{1+Bx_{2}}$, which imply that the prey growth rate and the predator death rate depend only on the prey supply. [8]. The other terms in (5) are the same as those in Equation (1).

By defining the new parameters

(6) $$\bar{a}=a-\frac{sc}{\bar{f}},\;\bar{f}=\frac{\bar{f}b}{\bar{b}+\bar{a}B},\;\bar{b}=b-\bar{a}B,$$

Model (5) can be expressed in the form of Equation (2) with different nonlinear functions:

(7) $$g_{1}\left(x_{1},x_{2}\right)=-Bx_{2}\frac{\bar{a}-\bar{b}x_{2}}{1+Bx_{2}},g_{2}\left(x_{1},x_{2}\right)=\frac{\bar{f}}{\bar{b}}Bx_{1}\frac{\bar{a}-\bar{b}x_{2}}{1+Bx_{2}}.$$

Some assumptions about the systems in **Case 1** and **Case 2** are needed for subsequent analysis. First, we consider the situation that the parameter s in the self-competition term for both cases is small. It describes the situation that the prey population density is small, and the impact of the self-competitions term $-sx_{1}^{2}$ is small. Second, we assume the term $Ax_{1}\ll 1$ for the **Case 1**. This assumption is consistent with the first one. Third, for **Case 2**, $Bx_{2}\ll 1$ is also necessarily needed to meet the requirement of the first assumption. For the convenience of following analysis, a small and positive parameter ε is introduced and the parameters s, A and B in Equation (2) are replaced by $\varepsilon^{2}s$, $\varepsilon^{2}A$, and $\varepsilon^{2}B$ to represent small values of these parameters. Thus, the mathematical model that we will investigate becomes

(8) $$\begin{array}{l} {\dot{x}}_{1}=x_{1}\left[\bar{a}-\bar{b}x_{2}-\frac{\varepsilon^{2}s}{\bar{f}}\left(-c+\bar{f}x_{1}\right)+\varepsilon^{2}g_{1}\left(x_{1},x_{2}\right)\right],\\ {\dot{x}}_{2}=x_{2}\left[-c+\bar{f}x_{1}+\varepsilon^{2}g_{2}\left(x_{1},x_{2}\right)\right]. \end{array}$$

with

(9) $$g_{1}\left(x_{1},x_{2}\right)=\frac{\bar{b}}{\bar{f}}Ax_{2}\frac{\bar{f}x_{1}-c}{1+\varepsilon^{2}Ax_{1}},g_{2}\left(x_{1},x_{2}\right)=-Ax_{1}\frac{\bar{f}x_{1}-c}{1+\varepsilon^{2}Ax_{1}},$$

for **Case 1** and with

(10) $$g_{1}\left(x_{1},x_{2}\right)=-Bx_{2}\frac{\bar{a}-\bar{b}x_{2}}{1+\varepsilon^{2}Bx_{2}},g_{2}\left(x_{1},x_{2}\right)=\frac{\bar{f}}{\bar{b}}Bx_{1}\frac{\bar{a}-\bar{b}x_{2}}{1+\varepsilon^{2}Bx_{2}}.$$

for **Case 2**.

### 2.2. The Stochastic Model

We take the random environmental fluctuations into account in Equation (8). Gaussian white noise and Poisson white noise are adopted to model the random environmental fluctuations, which cause random changes in the growth rate of the prey and death rate of the predators. This means that the growth rate of the prey and the death rate of the predators in Equation (8) are changed as:

(11) $$\bar{a}\to \bar{a}+\varepsilon \left(\zeta_{1}\left(t\right)+\xi_{1}\left(t\right)\right),\;c\to c+\varepsilon \left(\zeta_{2}\left(t\right)+\xi_{2}\left(t\right)\right),$$

Here, ε is the small parameter from Equation (8), which indicates that the random environmental influences are small. $\zeta_{i}\left(t\right)$$\left(i=1,2\right)$ are two independent Gaussian white noise excitations that are used to characterize the continuous environmental fluctuations. They have the following statistical characteristics:

(12) $$E\left[\zeta_{i}\left(t\right)\right]=0,\;E\left[\zeta_{i}\left(t+\tau \right)\zeta_{i}\left(t\right)\right]=2D_{i}\delta \left(\tau \right).$$

where E[•] represents the mathematical expectation and $2D_{i}$ are the noise intensities. Moreover, two independent Poisson white noises $\xi_{i}\left(t\right)$$\left(i=1,2\right)$ are used to model the jump effects in the environment, which can be viewed as the formal derivatives of compound Poisson processes $C_{i}\left(t\right)$

(13) $$\xi_{i}\left(t\right)=\frac{dC_{i}\left(t\right)}{dt},C_{i}\left(t\right)=\sum_{k=1}^{N_{i}\left(t\right)}Y_{ik}U\left(t-t_{ik}\right),\;i=1,2.$$

In Equation (14), $N_{i}\left(t\right)$ denote Poisson counting processes with mean arrival rates $\lambda_{i}>0$. $Y_{ik}$ represent the random magnitudes of the impulses that obey Gaussian distributions with variances $E[Y_{ik}^{2}]$ and zero mean in the present paper. U(•) is the unit step function. $\lambda_{i}E[Y_{ik}^{2}]$ are the intensities of the Poisson white noises. In addition, $\zeta_{i}\left(t\right)$ are also assumed to be independent with $\xi_{i}\left(t\right)$.

Consequently, the system that we will investigate can be described by the following Stratonovich stochastic differential equation (SDE) based on Equations (8) and (11),

(14) $$\begin{array}{l} {\dot{X}}_{1}=X_{1}\left[\bar{a}-\bar{b}X_{2}-\frac{\varepsilon^{2}s}{\bar{f}}\left(-c+\bar{f}X_{1}\right)+\varepsilon^{2}g_{1}\left(X_{1},X_{2}\right)+\varepsilon \zeta_{1}\left(t\right)+\varepsilon \xi_{1}\left(t\right)\right],\\ {\dot{X}}_{2}=X_{2}\left[-c+\bar{f}X_{1}+\varepsilon^{2}g_{2}\left(X_{1},X_{2}\right)+\varepsilon \zeta_{2}\left(t\right)+\varepsilon \xi_{2}\left(t\right)\right], \end{array}$$

where

(15) $$g_{1}\left(X_{1},X_{2}\right)=\frac{\bar{b}}{\bar{f}}AX_{2}\frac{\bar{f}X_{1}-c}{1+\varepsilon^{2}AX_{1}},g_{2}\left(X_{1},X_{2}\right)=-AX_{1}\frac{\bar{f}X_{1}-c}{1+\varepsilon^{2}AX_{1}},$$

for **Case 1** and

(16) $$g_{1}\left(X_{1},X_{2}\right)=-BX_{2}\frac{\bar{a}-\bar{b}X_{2}}{1+\varepsilon^{2}BX_{2}},g_{2}\left(X_{1},X_{2}\right)=\frac{\bar{f}}{\bar{b}}BX_{1}\frac{\bar{a}-\bar{b}X_{2}}{1+\varepsilon^{2}BX_{2}},$$

for **Case 2**. Mathematically, the symbols $X_{1}\left(t\right)$ and $X_{2}\left(t\right)$ are used to represent the stochastic processes corresponding to the deterministic functions $x_{1}\left(t\right)$ and $x_{2}\left(t\right)$ in Equation (8).

Equation (14) can be transferred to the following Itô SDE by adding some correction terms as follows [38]

(17) $$\begin{array}{ll} dX_{1} & =X_{1}\left[\bar{a}-\bar{b}X_{2}-\frac{s}{\bar{f}}\left(-c+\bar{f}X_{1}\right)+g_{1}\left(X_{1},X_{2}\right)+\varepsilon D_{1}\right]dt\\ & +\varepsilon \sqrt{2D_{1}}X_{1}dB_{1}\left(t\right)+X_{1}dC_{1}\left(t\right)+X_{1}\sum_{i=2}^{\infty }\frac{1}{i!}{\left(dC_{1}\left(t\right)\right)}^{i},\\ dX_{2} & =X_{2}\left[-c+\bar{f}X_{1}+g_{2}\left(X_{1},X_{2}\right)+\varepsilon D_{2}\right]dt+\varepsilon \sqrt{2D_{2}}X_{2}dB_{2}\left(t\right)\\ & +X_{2}dC_{2}\left(t\right)+X_{2}\sum_{i=2}^{\infty }\frac{1}{i!}{\left(dC_{2}\left(t\right)\right)}^{i}, \end{array}$$

in which $X_{1}\sum_{i=2}^{\infty }\frac{1}{i!}{\left(dC_{1}\left(t\right)\right)}^{i}$ and $X_{2}\sum_{i=2}^{\infty }\frac{1}{i!}{\left(dC_{2}\left(t\right)\right)}^{i}$ are two correction terms. We introduce the integral form of the compound Poisson process by Poisson random measures for further analysis [39]

(18) $$C_{k}\left(t\right)=\int_{0}^{t}\int_{Q_{k}}Y_{k}P_{k}\left(dt,dY_{k}\right)$$

where $P_{k}\left(dt,dY_{k}\right)$ are Poisson random measures and $Q_{k}$ are the Poisson mark spaces. Based on the Poisson random measure, the differentiation of compound Poisson processes can be written as

(19) $${\left(dC_{k}\left(t\right)\right)}^{i}=\int_{0}^{t}\int_{Q_{k}}Y_{k}^{i}P_{k}\left(dt,dY_{k}\right),i=1,2,\cdots$$

Then, the equivalent stochastic integro-differential equation (SIDE) of Equation (17) can be derived as [40]

(20) $$\begin{array}{ll} dX_{1} & =X_{1}\left[\bar{a}-\bar{b}X_{2}-\frac{\varepsilon^{2}s\left(-c+\bar{f}X_{1}\right)}{\bar{f}}+\varepsilon^{2}g_{1}\left(X_{1},X_{2}\right)+\varepsilon^{2}D_{1}\right]dt\\ & +\varepsilon \sqrt{2D_{1}}X_{1}dB_{1}\left(t\right)+\int_{Q_{1}}\gamma_{11}P_{1}\left(dt,dY_{1}\right),\\ dX_{2} & =X_{2}\left[-c+\bar{f}X_{1}+\varepsilon^{2}g_{2}\left(X_{1},X_{2}\right)+\varepsilon^{2}D_{2}\right]dt+\varepsilon \sqrt{2D_{2}}X_{2}dB_{2}\left(t\right)\\ & +\int_{Q_{2}}\gamma_{22}P_{2}\left(dt,dY_{2}\right), \end{array}$$

where

(21) $$\gamma_{11}=X_{1}\sum_{i=1}^{\infty }\frac{\varepsilon^{i}}{i!}Y_{1}^{i}\;\mathrm{and}\;\gamma_{22}=X_{2}\sum_{i=1}^{\infty }\frac{\varepsilon^{i}}{i!}Y_{2}^{i}.$$

In the remainder of this paper, the stochastic dynamics of the stochastic ecosystems will be analyzed based on Equation (20). Different from the deterministic system, the probability measures for the prey and predator population are needed to describe the random behavior of the system with random excitations. For the study of the stochastic problem, probability plays an important role. Thus, an important task here is to obtain the PDFs for both prey and predator population densities. In this paper, we focus on the long-term behavior of this stochastic model, namely, the stationary responses of Equation (14). To derive the stationary PDFs, we use a stochastic averaging method as described below.

### 2.3. The Stochastic Averaging

Without considering the random excitations and the nonlinear function $g_{i}\left(x_{1},x_{2}\right)$ (*I* = 1, 2) and letting the self-competition term be 0, Equation (20) reduces to the famous LV model

(22) $$\begin{array}{l} {\dot{x}}_{1}=x_{1}\left(\bar{a}-\bar{b}x_{2}\right),\\ {\dot{x}}_{2}=x_{2}\left(-c+\bar{f}x_{1}\right). \end{array}$$

It is known [1] that the system (22) has periodic trajectories that can be defined by

(23) $$r\left(x_{1},x_{2}\right)=\bar{f}x_{1}-c-c\ln\frac{\bar{f}x_{1}}{c}+\bar{b}x_{2}-\bar{a}-\bar{a}\ln\frac{\bar{b}x_{2}}{\bar{a}}=Q,$$

where Q is a constant and $r\left(x_{1},x_{2}\right)=0$ at both the unstable equilibrium point $\left(0,0\right)$ and the stable non-asymptotic equilibrium point $\left(\bar{c}/\bar{f},\bar{a}/\bar{b}\right)$ of Equation (22). This means that Equation (23) is the first integral of the system (22) for any positive value Q [1]. The period of the periodic trajectories with constant Q is determined by

(24) $$T\left(Q\right)=\oint dt=\oint \frac{dx_{2}}{x_{2}\left(\bar{f}x_{1}-c\right)}=\oint \frac{dx_{1}}{x_{1}\left(\bar{a}-\bar{b}x_{2}\right)}.$$

**Figure 1:**
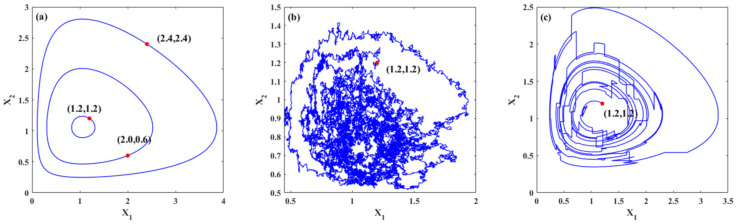
Typical trajectories of the systems. (**a**) Deterministic trajectories of system (22) with different initial values; (**b**) one trajectory of the system under Gaussian white noises only with initial value (1.2,1.2); (**c**) one trajectory of the system under Poisson white noises only with initial value (1.2,1.2).

Figure 1 demonstrates the trajectories of the system for three cases. Figure 1a shows the periodic trajectories for system (22) with different initial values. For different initial values, different closed orbits are obtained, namely, periodic solutions. Figure 1b,c show the random trajectories for system (22) under Gaussian white noise and Poisson white noise, respectively. It is known that stochastic excitation has a significant influence on the dynamics of the ecosystems. The trajectories of the system under stochastic excitations are random. Moreover, the trajectory changes with continuous slight jumps for the system with Gaussian white noises, while for the system excited by Poisson white noises, the trajectory includes some large jumps, which demonstrates the influences of different stochastic excitations on the trajectories. It can also be seen in these figures that large population densities of species may evolve into smaller population densities with the evolution of time. For an ecosystem, a smaller population density means that the population is more likely to die out, which means that the system is relatively fragile or unstable.

Replacing the variables $x_{1}$ and $x_{2}$ by stochastic processes $X_{1}$ and $X_{2}$ in Equation (23), the random counterpart of the first integral (23) can be obtained as

(25) $$R\left(t\right)=R\left(X_{1},X_{2}\right)=\bar{f}X_{1}-c-c\ln\frac{\bar{f}X_{1}}{c}+\bar{b}X_{2}-\bar{a}-\bar{a}\ln\frac{\bar{b}X_{2}}{\bar{a}}.$$

Based on the stochastic chain rule [39], the differential form of the first integral is given as


(26) $$\begin{array}{ll} dR\left(t\right) & =\left(\bar{f}X_{1}-c\right)\left[-\frac{\varepsilon^{2}s}{\bar{f}}\left(-c+\bar{f}X_{1}\right)+\varepsilon^{2}g_{1}\left(X_{1},X_{2}\right)+\varepsilon^{2}D_{1}+\varepsilon^{2}cD_{1}\right]dt\\ & +\left(\bar{b}X_{2}-\bar{a}\right)\left[\varepsilon^{2}g_{2}\left(X_{1},X_{2}\right)+\varepsilon^{2}D_{2}+\varepsilon^{2}aD_{2}\right]dt+\varepsilon \sqrt{2D_{1}}\left(\bar{f}X_{1}-c\right)dB_{1}\left(t\right)\\ & +\varepsilon \sqrt{2D_{2}}\left(\bar{b}X_{2}-\bar{a}\right)dB_{2}\left(t\right)+\int_{Q_{1}}\left[\bar{f}\gamma_{11}-c\ln\left(1+\frac{\gamma_{11}}{X_{1}}\right)\right]P_{1}\left(dt,dY_{1}\right)\\ & +\int_{Q_{2}}\left[\bar{b}\gamma_{22}-\bar{a}\ln\left(1+\frac{\gamma_{22}}{X_{2}}\right)\right]P_{2}\left(dt,dY_{2}\right). \end{array}$$

Taking the Taylor expansions of $\ln\left(1+\gamma_{11}/X_{1}\right)$ and $\ln\left(1+\gamma_{22}/X_{2}\right)$, and substituting them in Equation (26), one arrives at

(27) $$\begin{array}{ll} dR\left(t\right) & =\left(\bar{f}X_{1}-c\right)\left[-\frac{\varepsilon^{2}s}{\bar{f}}\left(-c+\bar{f}X_{1}\right)+\varepsilon^{2}g_{1}\left(X_{1},X_{2}\right)+\varepsilon^{2}D_{1}+\varepsilon^{2}cD_{1}\right]dt\\ & +\left(\bar{b}X_{2}-\bar{a}\right)\left[\varepsilon^{2}g_{2}\left(X_{1},X_{2}\right)+\varepsilon^{2}D_{2}+\varepsilon^{2}aD_{2}\right]dt\\ & +\varepsilon \sqrt{2D_{1}}\left(\bar{f}X_{1}-c\right)dB_{1}\left(t\right)+\varepsilon \sqrt{2D_{2}}\left(\bar{b}X_{2}-\bar{a}\right)dB_{2}\left(t\right)\\ & +\int_{Q_{1}}\left[\varepsilon A_{11}Y_{1}+\varepsilon^{2}A_{12}Y_{1}^{2}+\varepsilon^{3}A_{13}Y_{1}^{3}+\varepsilon^{4}A_{14}Y_{1}^{4}\text{+}\cdots \right]P_{1}\left(dt,dY_{1}\right)\\ & +\int_{Q_{2}}\left[\varepsilon A_{21}Y_{2}+\varepsilon^{2}A_{22}Y_{2}^{2}+\varepsilon^{3}A_{23}Y_{2}^{3}+\varepsilon^{4}A_{24}Y_{2}^{4}\text{+}\cdots \right]P_{2}\left(dt,dY_{2}\right). \end{array}$$

in which

$$A_{11}=\bar{f}X_{1}-c;\;A_{12}=\frac{\bar{f}X_{1}}{2};\;A_{13}=\frac{\bar{f}X_{1}}{6};\;A_{14}=\frac{\bar{f}X_{1}}{24};\;A_{21}=\bar{b}X_{2}-\bar{a};\;A_{22}=\frac{\bar{b}X_{2}}{2};\;A_{23}=\frac{\bar{b}X_{2}}{6};\;A_{24}=\frac{\bar{b}X_{2}}{24}.$$

Replacing $X_{1}$ in Equation (20) by $R\left(t\right)$ yields a new system governed by Equation (27) and the second equation of Equation (20). $R\left(t\right)$ is a slowly varying variable since ε is a small parameter, while $X_{i}$ are rapidly varying variables. Based on the stochastic averaging method proposed by [41,42], one can derive the averaged GFPK equation for $R\left(t\right)$ as

(28) $$\begin{array}{ll} \frac{\partial }{\partial t}p\left(r,t\right) & =-\frac{\partial }{\partial r}\left({\bar{A}}_{1}p\left(r,t\right)\right)+\frac{1}{2!}\frac{\partial^{2}}{\partial r^{2}}\left({\bar{A}}_{2}p\left(r,t\right)\right)-\frac{1}{3!}\frac{\partial^{3}}{\partial r^{3}}\left({\bar{A}}_{3}p\left(r,t\right)\right)\\ & +\frac{1}{4!}\frac{\partial^{4}}{\partial r^{4}}\left({\bar{A}}_{4}p\left(r,t\right)\right)+O\left(\varepsilon^{5}\right), \end{array}$$

where $p\left(r,t\right)$ is the PDF of $R\left(t\right)$ at time t. $O(\varepsilon^{5})$ represents the terms of $\varepsilon^{5}$ and higher, which contains an infinite number of terms of GFPK equation with small magnitude due to the increasing powers of ε. The other parameters of Equation (28) are given as

(29) $${\bar{A}}_{1}=\varepsilon^{2}{\bar{A}}_{11}+\varepsilon^{4}{\bar{A}}_{12},$$

(30) $${\bar{A}}_{11}={\langle U_{0}+A_{12}\lambda_{1}\left[Y_{1}^{2}\right]+A_{22}\lambda_{2}\left[Y_{2}^{2}\right]\rangle }_{t},$$

(31) $${\bar{A}}_{12}={\langle A_{14}\lambda_{1}\left[Y_{1}^{4}\right]+A_{24}\lambda_{2}\left[Y_{2}^{4}\right]\rangle }_{t},$$

(32) $$\begin{array}{c} U_{0}=\left(fX_{1}-c\right)\left[-\frac{s}{f}\left(-c+fX_{1}\right)+g_{1}\left(X_{1},X_{2}\right)+D_{1}\left(1+c\right)\right]\\ +\left(bX_{2}-a\right)\left[g_{2}\left(X_{1},X_{2}\right)+D_{2}\left(1+a\right)\right], \end{array}$$

(33) $${\bar{A}}_{2}=\varepsilon^{2}{\bar{A}}_{21}+\varepsilon^{4}{\bar{A}}_{22},$$

(34) $${\bar{A}}_{21}={\langle A_{11}^{2}\left(2D_{1}+\lambda_{1}E\left[Y_{1}^{2}\right]\right)+A_{21}^{2}\left(2D_{2}+\lambda_{2}E\left[Y_{2}^{2}\right]\right)\rangle }_{t},$$

(35) $${\bar{A}}_{22}={\langle \left(A_{12}^{2}+2A_{11}A_{13}\right)\lambda_{1}E\left[Y_{1}^{4}\right]+\left(A_{22}^{2}+2A_{21}A_{23}\right)\lambda_{2}E\left[Y_{2}^{4}\right]\rangle }_{t},$$

(36) $${\bar{A}}_{3}=\varepsilon^{4}{\bar{A}}_{31}=\varepsilon^{4}{\langle 3A_{11}^{2}A_{12}\lambda_{1}E\left[Y_{1}^{4}\right]+3A_{21}^{2}A_{22}\lambda_{2}E\left[Y_{2}^{4}\right]\rangle }_{t},$$

(37) $${\bar{A}}_{4}=\varepsilon^{4}{\bar{A}}_{41}=\varepsilon^{4}{\langle A_{11}^{4}\lambda_{1}E\left[Y_{1}^{4}\right]+A_{21}^{4}\lambda_{2}E\left[Y_{2}^{4}\right]\rangle }_{t},$$

where the symbol ${\langle \left[\;\right]\rangle }_{t}$ means the time average in one quasi-period, which is defined as

(38) $${\langle \left[\;\right]\rangle }_{t}=\frac{1}{T}\oint \left[\;\right]dt=\frac{1}{T}\oint \frac{\left[\;\right]dx_{2}}{x_{2}\left(fx_{1}-c\right)}=\frac{1}{T}\oint \frac{\left[\;\right]dx_{1}}{x_{1}\left(a-bx_{2}\right)},$$

where T is the period given in Equation (24).

It can be found from Equations (29)–(37) that the coefficients consist of terms of order $\varepsilon^{2}$ and $\varepsilon^{4}$. In the absence of terms of order $\varepsilon^{4}$ and higher in Equation (28) the averaged GFPK Equation (28) will reduce to the averaged FPK equation for the system under only Gaussian white noises $\zeta_{i}\left(t\right)$ with intensities $\left(2D_{i}+\lambda_{i}E[Y_{i}^{2}]\right)$(i=1,2). By solving this reduced FPK equation, one can obtain the Gaussian approximation solution for the averaged GFPK equations. However, the Gaussian approximation solution is not a proper approximation for small values of λ for Poisson white noise with the same noise intensity since the influence of term of order $\varepsilon^{4}$ in Equation (28) cannot be ignored. Therefore, more terms of the GFPK equations should be considered to obtain a more accurate solution when dealing with the system with Poisson white noise.

## 3. The Approximate Stationary Responses

The PDFs of the population densities are obtained by solving the averaged GFPK Equation (28) with certain boundary and initial conditions. In the present paper, only the long-term behaviors of the ecosystem are studied. A perturbation technique [43] is applied to derive the stationary PDFs $p_{R}\left(r\right)$ for $R\left(t\right)$ by solving the following reduced averaged GFPK equation

(39) $$\begin{array}{ll} 0 & =-\frac{\partial }{\partial r}\left({\bar{A}}_{1}p_{R}\left(r\right)\right)+\frac{1}{2!}\frac{\partial^{2}}{\partial r^{2}}\left({\bar{A}}_{2}p_{R}\left(r\right)\right)-\frac{1}{3!}\frac{\partial^{3}}{\partial r^{3}}\left({\bar{A}}_{3}p_{R}\left(r\right)\right)\\ & +\frac{1}{4!}\frac{\partial^{4}}{\partial r^{4}}\left({\bar{A}}_{4}p_{R}\left(r\right)\right)+O\left(\varepsilon^{5}\right), \end{array}$$

A second-order perturbation solution

(40) $$p_{R}\left(r\right)=p_{0}\left(r\right)+\varepsilon p_{1}\left(r\right)+\varepsilon^{2}p_{2}\left(r\right)$$

is adopted here to derive the approximate solution of Equation (39). By substituting Equation (40) into Equation (39) and putting terms of the same order of ε together, the equations for $p_{0}\left(r\right)$, $p_{1}\left(r\right)$, $p_{2}\left(r\right)$ are given as

(41) $$-\frac{\partial }{\partial r}\left(\varepsilon^{2}{\bar{A}}_{11}p_{0}\left(r\right)\right)+\frac{1}{2}\frac{\partial^{2}}{\partial r^{2}}\left(\varepsilon^{2}{\bar{A}}_{12}p_{0}\left(r\right)\right)=0$$

(42) $$-\frac{\partial }{\partial r}\left(\varepsilon^{3}{\bar{A}}_{11}p_{1}\left(r\right)\right)+\frac{1}{2}\frac{\partial^{2}}{\partial r^{2}}\left(\varepsilon^{3}{\bar{A}}_{12}p_{1}\left(r\right)\right)=0$$

(43) $$\begin{array}{l} -\frac{\partial }{\partial r}\left(\varepsilon^{4}{\bar{A}}_{11}p_{2}\left(r\right)\right)+\frac{1}{2}\frac{\partial^{2}}{\partial r^{2}}\left(\varepsilon^{4}{\bar{A}}_{12}p_{2}\left(r\right)\right)\\ =\frac{\partial }{\partial r}\left(\varepsilon^{4}{\bar{A}}_{12}p_{0}\left(r\right)\right)-\frac{1}{2}\frac{\partial^{2}}{\partial r^{2}}\left(\varepsilon^{4}{\bar{A}}_{22}p_{0}\left(r\right)\right)\\ +\frac{1}{3!}\frac{\partial^{3}}{\partial r^{3}}\left(\varepsilon^{4}{\bar{A}}_{31}p_{0}\left(r\right)\right)-\frac{1}{4!}\frac{\partial^{4}}{\partial r^{4}}\left(\varepsilon^{4}{\bar{A}}_{41}p_{0}\left(r\right)\right) \end{array}$$

It can be seen from Equation (41) that $p_{0}\left(r\right)$ is the solution for the system excited by Gaussian white noise with the same noise intensity, namely, approximate Gaussian solutions for the system excited by Poisson white noise. By solving Equations (41)–(43) step-by-step, the second-order perturbation solution (40) can be obtained. Then, the approximate stationary joint PDFs $p_{X_{1}X_{2}}\left(x_{1},x_{2}\right)$ take the form

(44) $$p_{X_{1}X_{2}}\left(x_{1},x_{2}\right)=\frac{p_{R}\left(r\right)}{x_{1}x_{2}T\left(r\right)}.$$

The marginal PDFs and moments of $X_{1}$ and $X_{2}$ can be calculated from Equation (44), such as

(45) $$p_{X_{1}}\left(x_{1}\right)=\int_{0}^{\infty }p_{X_{1}X_{2}}\left(x_{1},x_{2}\right)dx_{2},p_{X_{2}}\left(x_{2}\right)=\int_{0}^{\infty }p_{X_{1}X_{2}}\left(x_{1},x_{2}\right)dx_{1}$$

(46) $$E\left[X_{1}\right]=\int_{0}^{\infty }x_{1}p_{X_{1}}\left(x_{1}\right)dx_{1},\;Var\left(X_{1}\right)=\int_{0}^{\infty }{\left[x_{1}-E(X_{1})\right]}^{2}dx_{1}$$

(47) $$E\left[X_{2}\right]=\int_{0}^{\infty }x_{2}p_{X_{2}}\left(x_{2}\right)dx_{2},Var\left(X_{2}\right)=\int_{0}^{\infty }{\left[x_{2}-E(X_{2})\right]}^{2}dx_{2}$$

Furthermore, when the Poisson white noise intensity is 0, the method developed in this paper reduces to the case excited by Gaussian white noise. The effects of the Gaussian noise on the dynamics of the ecosystems have been studied by Cai [7]. Therefore, in the following subsections, we focus on the influence of the system parameters and Poisson white noise parameters on the stationary statistics.

### 3.1. The Influence of System Parameters

In this section, the influences of system parameters on the model (14) for **Case 1** and **Case 2** are shown in Figure 2, Figure 3, Figure 4, Figure 5, Figure 6, Figure 7, Figure 8 and Figure 9. The stochastic properties of population densities of the prey and predator can be calculated from Equations (44)–(47).

**Case 1:** prey is abundant compared with the predator

**Figure 2:**
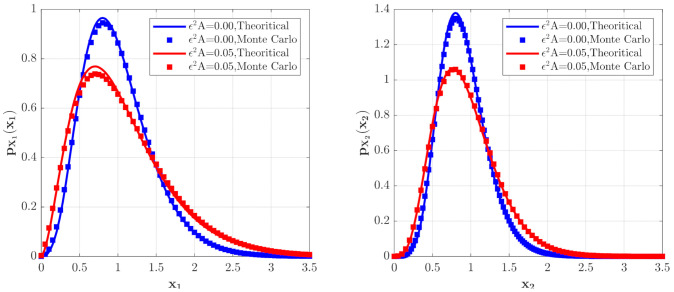
The PDFs of $X_{1}$ for the model with predator saturation term (**Case 1**) for different $\varepsilon^{2}A$ values.

The effects of the parameter $\varepsilon^{2}A$ in the predator saturation term of Equation (15) on the stationary PDFs of the species are shown in Figure 2. The PDF $p_{X_{1}}\left(x_{1}\right)$ of the population density of the prey $X_{1}\left(t\right)$ and the PDF of the population density of the predators $X_{2}$ are calculated with the following parameters a=1.0, b=1.0, c=0.5, f=0.5, $\varepsilon^{2}s=0.1$, $2\varepsilon^{2}D_{i}=0.005$, $\lambda_{i}=0.5$, $\varepsilon^{2}E[Y_{i}^{2}]=0.02$, i=1,2, and two different values of $\varepsilon^{2}A$. One can see from Figure 2 that the occurrence of the predator saturation influences the system behavior significantly. Compared with the case of $\varepsilon^{2}A=0.0$, the maxima of the PDFs of the species population densities become lower, and the probabilities for both small and large populations increase for $\varepsilon^{2}A=0.05$. This means that the ecosystem becomes more unstable for $\varepsilon^{2}A=0.05$. This is reasonable in the real world. Since the parameter $\varepsilon^{2}A$ represents the case of large prey population, it is obvious that the ecosystem is more unstable when this extreme case occurs. The solid lines in Figure 2 show the second-order perturbation solutions, while the discrete points denote the results obtained from Monte Carlo simulation. The good agreement between these two results shows the effectiveness of the second-order perturbation solution.

**Figure 3:**
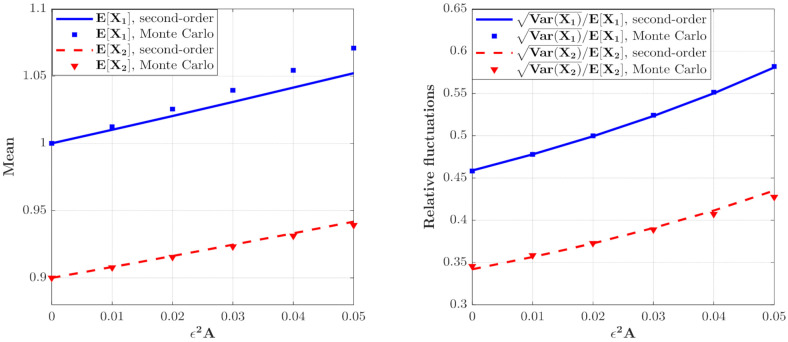
The means and relative fluctuations of $X_{1}$ and $X_{2}$ and $X_{2}$ for the model with predator saturation term (**Case 1**) for different $\varepsilon^{2}A$ value. The parameters are the same as those in Figure 2 except for $\lambda_{i}=1.0$, $E[Y_{i}^{2}]=0.01$, i=1,2.

The effect of $\varepsilon^{2}A$ on the moments of the population densities of prey and predators which can be calculated from Equations (46) and (47) are depicted in Figure 3. It can be seen that the curves for the means and relative fluctuations $\sqrt{Var\left(X_{i}\right)}/E\left[X_{i}\right]$ of the population densities of both species increase monotonously. This means that the ecosystem fluctuates more strongly for larger value of $\varepsilon^{2}A$, which implies a more unstable system.

**Case 2**: predator population is large

**Figure 4:**
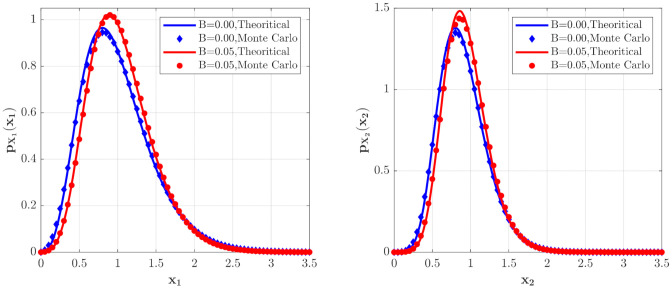
The PDFs of $X_{1}$ and $X_{2}$ for the model with the predator competition term (**Case 2**) for different $\varepsilon^{2}B$ values. The parameters are the same as those in Figure 2 except for $\lambda_{i}=0.5$, $\varepsilon^{2}E[Y_{i}^{2}]=0.02$, i=1,2.

**Figure 5:**
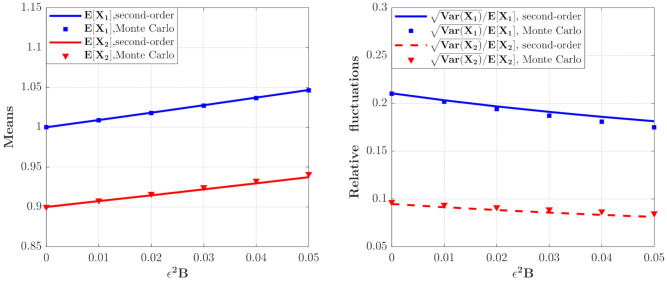
The means and relative fluctuations of $X_{1}$ and $X_{2}$ for the model with the predator competition term (**Case 2**) for different $\varepsilon^{2}B$ values. The parameters are the same as those in Figure 4 except $\lambda_{i}=1.0$, $\varepsilon^{2}E[Y_{i}^{2}]=0.01$, i=1,2.

Figure 4 depicts the PDFs of $X_{1}$ and $X_{2}$ for the model in **Case 2** for different $\varepsilon^{2}B$ values. It is found that the maxima of the PDFs for the case of $\varepsilon^{2}B=0.05$ are larger and the probability in this case is more concentrated. Figure 5 shows the mean and relative fluctuation of the species population of the ecosystem. It can be seen that the mean value of the prey and predator increase with increase of the $\varepsilon^{2}B$ value, while the relative fluctuation curves decrease with increasing the value of $\varepsilon^{2}B$, implying a more stable ecosystem. The results of the Monte Carlo simulations are also given in Figure 4 and Figure 5 to validate the results of the proposed method.

### 3.2. The Influence of Poisson White Noise

In this section, we focus on the influences of the parameters of the Poisson white noises, namely mean arrival rate λ and the variance of amplitude $E[Y^{2}]$.

**Figure 6:**
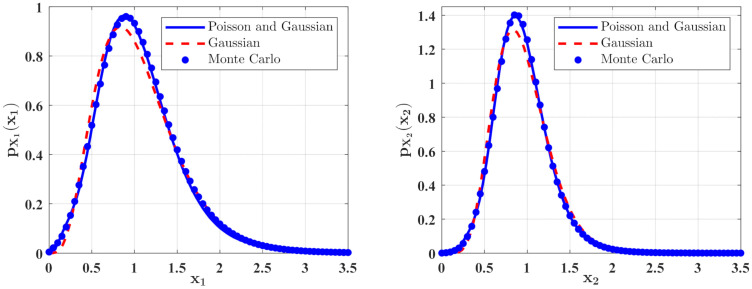
The PDFs of $X_{1}$ and $X_{2}$ for **Case 1**. The parameters are the same as those in Figure 2 except for $\varepsilon^{2}A=0.05$, $2\varepsilon^{2}D_{i}=0.001$, $\lambda =\lambda_{i}=0.1$, $\varepsilon^{2}E[Y^{2}]=\varepsilon^{2}E[Y_{i}^{2}]=0.08$, i=1,2.

**Figure 7:**
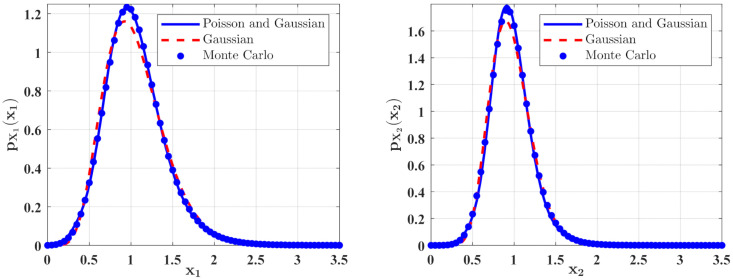
The PDFs of $X_{1}$ and $X_{2}$ for **Case 2**. The parameters are the same as those in Figure 4 except for $\varepsilon^{2}s=0.08$, $\varepsilon^{2}B=0.05$, $2\varepsilon^{2}D_{i}=0.001$, $\lambda =\lambda_{i}=0.2$, $\varepsilon^{2}E[Y^{2}]=\varepsilon^{2}E[Y_{i}^{2}]=0.04$, i=1,2.

In Figure 6 and Figure 7, some numerical calculations have been performed to obtain the PDFs of predator and prey population densities in the model (14) for **Case 1** and **Case 2**, respectively. In these figures, the second-order perturbation results are represented by the solid lines. The dashed lines are the approximate Gaussian solutions. The Monte Carlo results are plotted as dotted lines. It is found that the PDFs obtained by the present method are closer to the Monte Carlo simulation than the approximate Gaussian solutions. The maxima of the stationary PDFs for the system with both Gaussian and Poisson white noise are higher than for the approximate Gaussian solutions. This means that the second-order perturbation solutions have higher accuracy than the Gaussian approximation solution.

**Figure 8:**
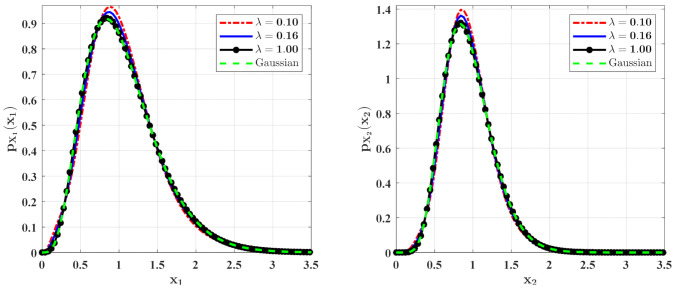
The PDFs of $X_{1}$ and $X_{2}$ for **Case 1** with different mean arrival rate λ. The other parameters are the same as those in Figure 6.

**Figure 9:**
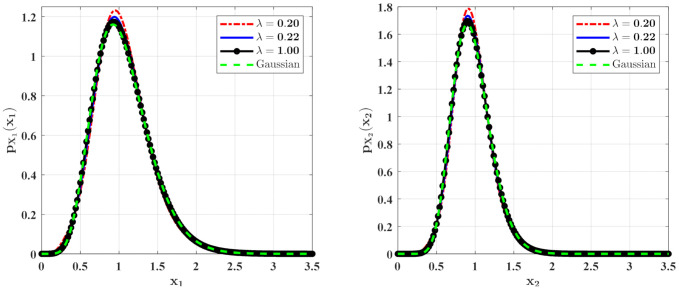
The PDFs of $X_{1}$ and $X_{2}$ for **Case 2** with different mean arrival rate λ. The other parameters are the same as those in Figure 7.

Figure 8 and Figure 9 show the influences of λ of the Poisson white noises on the stationary PDFs when the Poisson white noise intensity $\lambda E[Y^{2}]$ is kept constant. It is observed from these figures that with the increase of λ, the maxima of the PDFs decrease and the PDFs for population densities of prey and predator approach the ones for the system with the same intensity of Gaussian white noise excitation. To see this clearly, we define the $L_{2}$ error norm between the PDFs for the system with Poisson white noise and the Gaussian approximation solution as

(48) $$l2\left(x_{i}\right)=\int_{0}^{+\infty }{\left(p_{X_{i}}\left(x_{i}\right)-p_{x_{i}}^{G}\left(x_{i}\right)\right)}^{2}dx_{i},\;i=1,2$$

where $p_{X_{i}}\left(x_{i}\right)$ are the second-order perturbation PDFs for $X_{i}$ and $p_{x_{i}}^{G}\left(x_{i}\right)$ are the Gaussian approximation solutions. We calculate the errors for different λ for **Case 1** (Table 1) and **Case 2** (Table 2). It can also be seen from these tables that with the increase of λ, the error decreases significantly, which also verifies our above conclusion.

## 4. Conclusions

In the present paper, we have investigated the statistical responses of a stochastic prey–predator type model for the possible situations of sufficient prey supply and a large predator population. The statistical responses of the species population, including the approximate stationarity PDFs and moments, have been obtained by a stochastic averaging and a perturbation technique. It can be found that, for the system with abundant prey, the increase of the parameter $\varepsilon^{2}A$ describing the saturation of the predator population from 0 to 0.5 leads to the increase of population fluctuations, which implies that the system becomes unstable. However, for the system where the predator population is large, the increase of the parameter $\varepsilon^{2}B$ describing the competition of predators for prey has the opposite effect. In addition, we have paid special attention to the effect of the mean arrival rate λ of the Poisson white noise. The influence of Poisson white noise on the system tends to approach the influence of Gaussian white noise with the same noise intensity when the λ increases from 0.05 to 2.0.

Although only two types of ecosystems with combined Gaussian and Poisson white noise have been investigated in this paper, our approaches can be applied to other ecosystem models. Except for the stochastic response of the ecosystem being investigated, the stochastic optimal control or extinction problem are also worth studying.

## Figures and Tables

**Table 1:** Error $l2\left(x_{i}\right)$ for PDFs in **Case 1**.

λ	0.05	0.08	0.2	0.4	0.6	0.8	1.0	2.0
*l*2(*x*_1_)	1.19 × 10^−2^	7.61 × 10^−3^	9.90 × 10^−4^	4.52 × 10^−4^	1.03 × 10^−4^	3.22 × 10^−5^	1.84 × 10^−5^	9.37 × 10^−8^
*l*2(*x*_2_)	1.54 × 10^−2^	9.78 × 10^−3^	1.27 × 10^−3^	5.85 × 10^−4^	1.33 × 10^−4^	4.10 × 10^−5^	2.34 × 10^−5^	1.28 × 10^−7^

**Table 2:** Error $l2\left(x_{i}\right)$ for PDFs in **Case 2**.

λ	0.05	0.08	0.2	0.4	0.6	0.8	1.0	2.0
*l*2(*x*_1_)	4.39 × 10^−2^	1.78 × 10^−2^	4.20 × 10^−3^	1.21 × 10^−3^	6.72 × 10^−4^	1.10 × 10^−4^	3.81 × 10^−5^	3.05 × 10^−7^
*l*2(*x*_2_)	6.05 × 10^−2^	2.44 × 10^−2^	5.72 × 10^−3^	1.65 × 10^−3^	9.29 × 10^−4^	1.50 × 10^−4^	5.19 × 10^−5^	4.21 × 10^−7^

## Data Availability

Not applicable.
